# Shear Stress-Induced Alteration of Epithelial Organization in Human Renal Tubular Cells

**DOI:** 10.1371/journal.pone.0131416

**Published:** 2015-07-06

**Authors:** Damien Maggiorani, Romain Dissard, Marcy Belloy, Jean-Sébastien Saulnier-Blache, Audrey Casemayou, Laure Ducasse, Sandra Grès, Julie Bellière, Cécile Caubet, Jean-Loup Bascands, Joost P. Schanstra, Bénédicte Buffin-Meyer

**Affiliations:** 1 Institut National de la Santé et de la Recherche Médicale (INSERM), U1048, Toulouse, France; 2 Université Toulouse III Paul Sabatier, Institute of Metabolic and Cardiovascular Diseases - I2MC, Toulouse, France; National Cancer Institute, UNITED STATES

## Abstract

Tubular epithelial cells in the kidney are continuously exposed to urinary fluid shear stress (FSS) generated by urine movement and recent *in vitro* studies suggest that changes of FSS could contribute to kidney injury. However it is unclear whether FSS alters the epithelial characteristics of the renal tubule. Here, we evaluated *in vitro* and *in vivo* the influence of FSS on epithelial characteristics of renal proximal tubular cells taking the organization of junctional complexes and the presence of the primary cilium as markers of epithelial phenotype. Human tubular cells (HK-2) were subjected to FSS (0.5 Pa) for 48h. Control cells were maintained under static conditions. Markers of tight junctions (Claudin-2, ZO-1), Par polarity complex (Pard6), adherens junctions (E-Cadherin, β-Catenin) and the primary cilium (α-acetylated Tubulin) were analysed by quantitative PCR, Western blot or immunocytochemistry. In response to FSS, Claudin-2 disappeared and ZO-1 displayed punctuated and discontinuous staining in the plasma membrane. Expression of Pard6 was also decreased. Moreover, E-Cadherin abundance was decreased, while its major repressors Snail1 and Snail2 were overexpressed, and β-Catenin staining was disrupted along the cell periphery. Finally, FSS subjected-cells exhibited disappeared primary cilium. Results were confirmed *in vivo* in a uninephrectomy (8 months) mouse model where increased FSS induced by adaptive hyperfiltration in remnant kidney was accompanied by both decreased epithelial gene expression including ZO-1, E-cadherin and β-Catenin and disappearance of tubular cilia. In conclusion, these results show that proximal tubular cells lose an important number of their epithelial characteristics after long term exposure to FSS both *in vitro* and *in vivo*. Thus, the changes in urinary FSS associated with nephropathies should be considered as potential insults for tubular cells leading to disorganization of the tubular epithelium.

## Introduction

Urinary fluid shear stress (FSS) is the friction force resulting from movement of urine on the surface of renal tubular cells. It depends on the urine flow rate and viscosity as well as on the diameter of the renal tubule.

Urinary FSS controls tubular exchanges (reabsorption and secretion) by modifying the activity and abundance of transport proteins [1–5]. Interestingly, there is a growing body of evidence showing that modified urinary FSS contributes to the progression of chronic kidney disease (CKD). First, urinary FSS is presumably changed in most of nephropathies, mainly because of variations of urinary flow and/ or viscosity (after renal mass reduction or in obstructive, diabetic or hypertensive nephropathies) [6–9], and the detection of urinary FSS is abolished or aberrant in polycystic kidneys resulting from mutations in genes encoding polycystins [10, 11]. Secondly, *in vitro* experiments on renal tubular cells showed that FSS targets a number of molecules involved in the development of CKD. For example, FSS inhibits the activity of plasminogen activators in proximal tubular cells [7, 12]. FSS also induces externalization of angiotensin II receptors from apical recycling endosomes to the apical plasma membrane in tubular cells [13]. A recent study from our laboratory showed that changes in FSS on proximal tubular cells induced upregulation of tubular damage markers such as Kidney injury molecule 1 and Neutrophil gelatinase-associated lipocalin [14]. FSS-injured cells also secrete mediators that stimulate adhesion of monocytes to endothelial cells and their differentiation into inflammatory macrophages [14, 15] suggesting that FSS acts *in vivo* as a promoter of renal inflammation. This combined body of evidence suggests that changes in urinary FSS potentially represent an early aggression for renal tubule cells, thereby playing a role in the progression of CKD [6].

Tubular function is determined by organization of renal tubule in a highly structured monolayer epithelium composed of polarized cells linked together by intercellular junctional complexes. The cell polarity results in the division of the plasma membrane into two distinct areas that differ by composition in proteins and lipids and by the presence of a primary cilium at the apical pole where it acts as a sensory organelle [16]. Tight junctions are formed of transmembrane proteins, including claudins, which interact with homolog proteins in the neighboring cells and with many cytoplasmic proteins such as zonula occludens proteins [17–19]. They provide the apicobasal polarity of tubular cells and regulate the paracellular flux of molecules between urine and interstitium. Adherens junctions are composed of transmembrane proteins, cadherins, which mediate ligation with cadherins on adjacent cells and interact with intracellular anchor proteins including catenins [20, 21]. Their role is to connect the adjacent cell cytoskeleton to form a cohesive epithelium. The renal tubule is recognized as a major target of both acute kidney injury and CKD [18, 19] and tubular lesions were observed in many pathophysiological states where modification of urinary FSS is suspected. For example, after reduction of renal mass (during nephrectomy in animal models or following cancers or trauma in human), chronic, compensatory, increased glomerular filtration rate (GFR) in residual nephrons [22–24] and epithelial tubular structural changes were observed [25, 26]. In early and poorly controlled diabetes, renal hyperfiltration may constitute a risk factor for the development of diabetic nephropathy [27–29]. In addition, in this context, early alterations in epithelial characteristics of the tubular wall were detected [30]. Given that increased GFR can lead to elevated urinary FSS and that previous data suggest the involvement of FSS in tubular aggression in nephropathies, we hypothesized that long term increase in FSS can contribute to the disorganization of the epithelial architecture of the renal tubule in CKD.

Here, we evaluated *in vitro* and *in vivo* the influence of FSS on epithelial characteristics of renal proximal tubular cells taking the organization of tight and adherens junctions and the presence of the primary cilium as markers of the epithelial phenotype. We also investigated the consequences of FSS-induced loss of epithelial phenotype on tubular cell death and acquisition of mesenchymal characteristics.

## Materials and Methods

### Cells

The human proximal tubule epithelial cell-line HK-2 [31] was purchased from American Type Tissue Culture. HK-2 cells were cultured at 37°C in 5% CO_2_ atmosphere in epithelial medium containing a 1/1 mix of Dulbecco's Modified Eagle Medium (DMEM, 11966 Gibco) and Ham’s F-12 Nutrient Mix (F-12, 21765 Gibco), Penicillin100 U/mL, Streptomycin 100 μg/mL, Hydrocortisone 36 ng/mL, Epidermal growth factor 10 ng/mL, Triiodotyronine 4 pg/mL, Insulin 5 μg/mL and supplemented with fetal bovine serum 10% (FBS, 10270 Gibco).

### Animals

Twelve male C57BL/6 mice were purchased at Charles River Laboratory. Mice were housed 4 per cage and maintained on a 12h light/12h dark cycle in a pathogen-free environment with free access to water and fed ad libitum a regular diet (D12450B Research Diet) for the duration of the protocol. At the age of 6 weeks, six mice underwent uninephrectomy (UNx) or were sham-operated (sham). Briefly, mice were anesthetized under a flux of oxygen/isoflurane (3%) mix. A dorsal incision was practiced to expose the right kidney and the lower renal artery branch was ligatured and sectioned. Muscle and cutaneous layers were sutured with 2 pints each and isoflurane flux was stopped. An injection of buprenorphine (100 μg/kg, sc) is performed to minimize pain on awakening. Sham-operated mice underwent only dorsal incision and sutures. Sham- and UNx-mice were housed for 8 months before sacrifice and collection of the kidney and blood for further analysis. All experiments were conducted in accordance with the NIH guide for the care and use of laboratory animals and were approved by the animal care and use committee from UMS US006/INSERM, Toulouse, France (protocol agreement #CEEA-122 2014–13).

### HK-2 Cell Exposure to Shear Stress

HK-2 cells were grown until confluence on plastic slides coated with collagen IV (20μg/mL, C5533 Sigma). Then slides were assembled into a home-made parallel plate flow chamber. A flask containing 20 mL epithelial medium supplemented with FBS 5% was connected to the input of the chamber. A peristaltic pump was connected on the side to the flask and on the other side to the output of the chamber. Flow of medium culture was controlled by peristaltic pump for 48h as indicated in results. The flow system was kept at 37°C in CO_2_ 5%. FSS was based on the formula 6 μQ/h^2^l where μ is the fluid dynamic viscosity (0.7x10^-3^Pa.s), Q is the flow rate (3 mL/min), h and l are respectively the flow channel thickness (0.205 mm) and width (10 mm). Estimations based on micropuncture studies in rat proximal tubules [32] have concluded that physiological FSS is about 0.1 Pa in the initial portion of the proximal tubule [5, 14, 15, 33]. In this study, a higher intensity (0.5 Pa) was used, as it is considered to mimic pathologic glomerular hyperfiltration [22, 23]. For the control condition (FSS 0), HK-2 cells were handled similarly but maintained in static conditions with 20mL of epithelial medium-FBS 5% for 48h, as HK-2 cells exposed to FSS.

### Analysis of mRNA Expression

Total RNAs were isolated from HK-2 cells or mice kidneys using the RNeasy plus Mini kit (Qiagen). RNA content and purity was quantified by a NanoDrop instrument (ND-1000 spectrophotometer, Thermo Fischer Scientific). cDNA was synthesized from 500ng of RNA using the SuperScript II Reverse Transcriptase kit (Invitrogen). Real-time PCR was performed with 12.5 ng of cDNA, 300 nM of forward and reverse primers (Integrated DNA Technologies, Table 1) using the SsoFast EvaGreen Supermix kit (Bio-Rad) and a StepOnePlus Real-Time PCR System (Applied Biosystems). Relative mRNA expression was calculated by the comparative Ct method (2^− ΔΔCt^) using GAPDH as reference gene. Primers used for PCR were the following: Cdc42 (F) GCATGCTTGTGGATGACTCTGT (R) TGGAACCAGGGAGCAGCTT; Collagen I (F) CCCCTGGAAAGAATGGAGATG (R) TCCAAACCACTGAAACCTCTG; E-Cadherin (F) CCCTCGACACCCGATTCA (R) CCAGGCGTAGACCAAGAAATG; N-Cadherin (F) AGGGGACCTTTTCCTCAAGA (R) CCGAGATGGGGTTGATAATG; GAPDH (F) CATGAGAAGTATGACAACAG (R) AGTCCTTCCACGATACCAAAGT; Fibronectin (F) TAGGCTTTGGAAGTGGTCATTTC (R) CAGCTCATCATCTGCCCATTT; Pard3 (F) AATCCCACACGCTGGTCAAC (R) TAGGACTCCCAGCAGTGTTCTG; Pard6 (F) GCGCAGTCCCGATAGCAT (R) TCGGAACTCGGCGTCAA; aPKC (F) CGTGGACACGCCTGATTG (R) CAGCCTGGCATGCATATGC; αSMA (F) CCGGGAGAAAATGACTCAAA (R) GCGTCCAGAGGCATAGAGAG; Snail1 (F) AGGTGGGCCTGGTCGTAG (R) CCCAATCGGAAGCCTAATTA; Snail2 (F) ACGCCCAGCTACCCAATG (R) TCACTCGCCCCAAAGATGAG; Vimentin (F) CCAGAGGGAGTGAATCCAGA (R) AGATGGCCCTTGACATTGAG.

**Table 1:** *In vitro* studies assessing the effects of FSS on epithelial characteristics of renal cells.

Cell	Culture cell substrate	FSS intensity	FSS duration	Cell characteristics in response of FSS	Ref
Mouse primary proximal cells	glass slide	0.01–0.07 Pa	24h	↓ cytosolic actin stress fibers ↑ lateral actin network	[7]
LLC-PK1 cell line	glass slide	0.01 Pa	24h	↑ lateral actin network	[7]
Mouse podocyte cell line	collagen IV coated glass slide	0.001–0.05 Pa	20h	↓ cytosolic actin stress fibers ↑ lateral α-Actinin (AJ) staining ≠ lateral ZO-1 (TJ) staining morphology: from continuous to discontinuous/punctuated	[46]
Mouse proximal tubular cell line	collagen coated glass slide	0.1 Pa	5h	↓ cytosolic actin stress fibers ↑ lateral actin network ≠ E-Cadherin (AJ) staining: from intracellular to lateral position ↑ lateral ZO-1 staining	[33]
Rat primary inner medullary collecting duct cells	porous fibronectin coated membrane	0.1 Pa	5h	↓ cytosolic actin stress fibers ↑ lateral actin network ≠ E-Cadherin staining: from intracellular to lateral position ↓ intracellular punctuated and ↑ lateral continuous β-Catenin (AJ) staining	[44]
Rat primary inner medullary collecting duct cells	glass slide	0.1 Pa	5h	↓ cytosolic actin stress fibers ↑ lateral actin network ↓ intracellular punctuated and ↑ lateral continuous β-Catenin staining	[44]
Human primary tubular cells^1^	supramolecular polymer mesh^2^	? ^4^	19 days	↑ lateral ZO-1 staining	[42]
Human primary tubular cells^1^	bioactive supramolecular polymer mesh^3^	? ^4^	19 days	≠ lateral ZO-1 staining morphology: from “straight” to “zig zag”/jagged	[42]
Rat primary inner medullary collecting duct cells	porous polyester membrane	0.1 Pa	3h	↓ cytosolic actin stress fibers ↑ lateral actin network	[2]
MDCK cell line	Thermanox slides	0.2 Pa	6h	↓ cytosolic actin stress fibers ≠ lateral actin network: clustering around cell edges ≠ lateral ZO-1 staining morphology: from “zig zag”/jagged to “straight”	[41]
HK-2 cell line	blank substrat^5^	0.002–0.1 Pa	2h	→ lateral ZO-1 staining (discontinuous/punctuated)	[45]
HK-2 cell line	topographical substrat^6^	0.002–0.1 Pa	2h	↑ lateral ZO-1 staining and ≠ staining morphology: more continuous/less punctuated	[45]
Human primary proximal tubular cells	porous ECM coated membrane	0.02 Pa	18h	↓ cytosolic actin stress fibers ≠ lateral ZO-1 staining morphology: from slight punctuated to continuous ↑ primary cilia formation	[43]
Murine immortalized cortical collecting duct cells	glass slide	0.02 Pa	30 min	↑ cytosolic actin stress fibers → lateral actin network → lateral Occludin (TJ) staining → lateral ZO-1 staining	[52]

FSS was tested on renal cells and changes in organization of actin cytoskeleton, adherens junctions (AJ), tight junctions (TJ) or primary cilium were determined by comparison with no FSS condition, where cells remained in static media durig the same time than flow tests.

Lateral, at the periphery of the cells; ECM, extracellular matrix; **↑**, increase in; ↓, decrease in; ≠, change in; → no change in.

^1^The population of tubular cells is heterogeneous because composed of cells derived from all tubular segments.

^2,3^The supramolecular polymers used were self-assembled into nano-meter scale fibers by electro-spinning to mimic basement membrane; bioactivity was introduced into these nano-fibers by intercalation of different ECM peptides, in order to ameliorate synthetic basement membrane.

^4^FSS induction was performed through flow of medium culture at a rate of 1 mL/h. However, without knowing the size of perfusion chamber, it is impossible to give the intensity of FSS.

^5,6^Topographically patterned substrate mimics micro- and nano-scale topographic structures contained in the basement membrane of the kidney tubule; blank substrates lacked of geometric structures.

### Western Blot Analysis

For protein extraction, HK-2 cells were lysed on ice in lysis buffer (Tris 10mM à pH 7.5, NaCl 150mM, EDTA 1mM, EGTA 1mM, SDS 0.1%, NP40 1% and deoxycholate 1%) supplemented with a protease inhibitor cocktail (Complete, Mini, Roche). Proteins were separated in a 4–15% SDS-PAGE and blotted on a nitrocellulose membrane (Amersham). Membranes were blocked with TBS-Tween 0.1% supplemented with milk 5% for 30 min. Then, membranes were incubated with primary antibodies (Anti E-Cadherin [1:200, Sc-7870 Santa Cruz], anti Fibronectin [1:500, F3648 Sigma], anti αSMA [1:500, Ab5964 AbCam], anti Vimentin [1:1000, MS-129-P Neomarkers], anti β-Actin (1:5000, A5441 Sigma)) overnight at 4°C followed by incubation with the peroxidase-conjugated secondary antibody (anti rabbit or anti mouse [1:10000, Bethyl]) for 1h. Specific bands were detected using SuperSignal West Pico Chemiluminescent Substrate (Thermo Scientific) and the ChemiDoc XRS+ System (Bio-Rad). Protein bands were quantified by densitometry using Image Lab software (version 4.0.1, Bio-Rad) and results are expressed as ratio between target protein and β-Actin.

### Immunocytochemistry

HK-2 cells were fixed with PBS-paraformaldehyde 4% for 15min, permeabilized with PBS-Triton X100 0.3% for 5 min and blocked with PBS-BSA 5% for 1h. Then cells were labeled with primary antibodies (anti β-Catenin [1:100 Sc-7963 Santacruz], Anti Claudin-2 [1:100 51–6100 Invitrogen], anti α-acetylated Tubulin [1:8000, T7451 Sigma] or anti ZO-1 [1:100 339100 Invitrogen]) for 1 h followed by a 1-h incubation with anti-mouse or anti-rabbit Alexa Fluor 488 or 548 secondary antibody (1:200, Invitrogen). To visualize actin filaments, Phalloidin-FluoProbes 547H (1:40, Interchim) was added instead antibodies. The slides were then covered with Vectashield mounting medium containing DAPI (Vector Laboratories). Fluorescence images were acquired with an Axio Observer Z.1m inverted microscope (Carl Zeiss) equipped with apotome system to image optical sections. In order to optimize the detection of immunostaining, exposure times are adapted to each photograph. The labeling intensities are thus not comparable from one image to another.

### Detection of Apoptosis and Necrosis

HK-2 cells were assessed for apoptosis and necrosis using Cell Meter Annexin V Binding Apoptosis Assay Kit (AAT Bioquest), according to the manufacturer's instructions. For this, cells were detached from slides using trypsin and then trypsin was inactivated with epithelial medium supplemented with FBS 10%. After gently centrifugation and washing with PBS, Annexin V-iFluor 488 (1:400) and Iodure propidium (1:400) were added to the cell suspension for incubation in the dark for 15 min at 37°C. Then, 0.3 ml of ice-cold 1× annexin-binding buffer was added, and induction analysis was conducted by flow cytometry (BD FACSVerse, BD Biosciences) with BD FacsSuite software (BD Biosciences) using FL1 and FL2 ranges for Annexin V-iFluor 488 and Iodure propidium, respectively. In each of the graphs, the bottom left quadrant represents live cells, the bottom right quadrant represents cells in early apoptosis and the top right quadrant represents cells in necrosis (primary or secondary).

### Evaluation of Renal Function

Glomerular filtration rate (GFR) was evaluated by measurement of FITC-inulin clearance, as previously described [34, 35]. Briefly, mice were anesthetized under a flux of oxygen/isoflurane (97/3%) before injecting retroorbitally 2 μl/g of a 5% solution of FITC-inulin (F3272 Sigma). Caudal blood (25–30 μl) was collected in heparinized tubes (Microvette 16.443 Sarstedt Nümbrecht) at 3, 7, 10, 15, 35, 55, 75 minutes post-injection of FITC-inulin. Isoflurane flux was then removed and the mice were replaced in their cage with free access to water and food. After blood centrifugation, FITC-inulin concentration was determined by spectrofluorimetry (485 nm excitation/538 nm emission) against a standard curve of FITC-inulin (0–100 μg/ml). GFR (μl/min) was calculated using a two-compartment clearance model (http://curvefit.com/id205.htm) as previously described [35]. The single kidney GFR (skGFR) value in sham mice was further estimated by urinary GFR/2 ratio.

### Renal Histology and Immunohistochemistry

Kidneys were collected from each mouse included in the renal function study. Tissues were fixed in Carnoy’s solution for 24 h, embedded in paraffin and micrometer sections were cut. For histological analysis, renal sections were subjected to periodic acid-Schiff staining (PAS), scanned using a Nanozoomer 2.0 RS (Hamamatsu Photonics SARL) and treated with the Morpho-expert image-analysis software (version 1.00, Explora Nova) for renal corpuscle surface quantification. For immunohistochemistry analysis of primary cilia, renal sections were deparaffinized in toluene and rehydrated from xylene through a series of graded ethanol washes. After treatment with sodium borohydride 1% (71321 Sigma, 20 min at 4°C) and washes in PBS—Tween 0.01%, sections were permeabilized in PBS—Triton X-100 0.2% for 20 min and blocked with PBS—BSA 3% for 30 min. Sections were then labeled with specific anti α-acetylated Tubulin antibody [1:200, T7451 Sigma] overnight at 4°C followed by a 1-h incubation with anti-mouse Alexa Fluor 568 secondary antibody (1:200, Invitrogen) supplemented with OG488-conjugated wheat germ agglutinin (WGA, Invitrogen). After covering of the slides with Vectashield mounting medium containing DAPI (Vector Laboratories), sections were scanned with a Nanozoomer 2.0 RS. For assessment of disappearance of cilia, the number of primary cilia in cortex renal tubules was manually counted using the NDP. view software and expressed as the number of tubular primary cilia per mm^2^ of renal area.

### Urine Analysis

Mice were placed in metabolic cages and urine was collected on a 24-h period. Urinary albumin concentration was determined with a mouse antigen specific ELISA (Mouse Albumin Quantitation Albuwell Kit, Exocell). Urinary creatinine concentration was determined using enzymatic colorimetric assay (Quantichrom Creatinine Assay Kit, BioAssay Systems).

### Statistical Analysis

Statistical analyses were performed using GraphPad Prism 5.0 for Windows (GraphPad Software Inc) and a Mann-Whitney’s test was performed to compare two groups (FSS 0.5 Pa *versus* FSS 0 [4–7 experiments] or UNx *versus* sham [6 mice per group]). All data are expressed as mean ± SEM. p<0.05 was considered as statistically significant.

## Results

### In Vitro Effect of Chronic High FSS on Intercellular Junctions of Renal Tubular Cells

**Fig 1:**
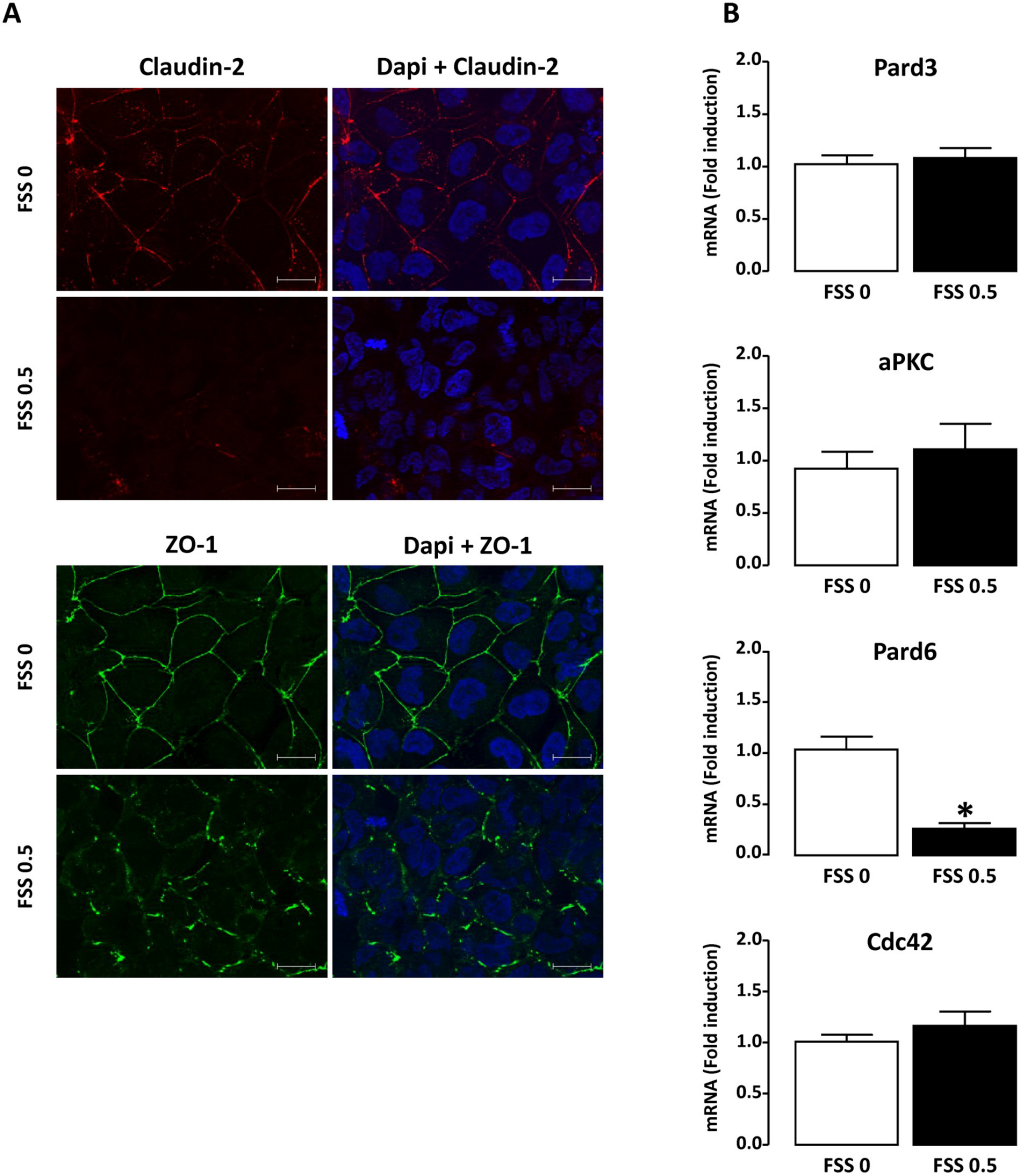
FSS induces disruption of tight junctions in renal tubular cells. Confluent monolayers of HK-2 cells were submitted to FSS 0 (static) or FSS 0.5 Pa (FSS 0.5) for 48h. **A**/ The localization of Claudin-2 or ZO-1 was analyzed by immunofluorescence. Cells were counterstained with DAPI. Pictures display representative areas of staining from three independent experiments. Red, Claudin-2; green, ZO-1; blue, DAPI-nuclei. Bars indicate 20 μm. **B/** The level of mRNA encoding for Pard3, Pard6, aPKC and Cdc42 was quantified by real-time PCR and results are expressed as the fold induction compared to static condition. Data represent mean ± SEM of 4–6 experiments. *p<0.01 *versus* FSS 0.

First, we examined the effect of FSS on tight and adherens junctions in tubular cells. For this, HK-2 tubular proximal cells were exposed to chronic and high FSS (0.5 Pa for 48h, see materials and methods) and were compared to cells maintained under static conditions. The organization of the tight junctions was evaluated by immunocytochemical analysis of the cellular distribution of two major proteins, Claudin-2 and ZO-1 (Zonula occludens-1). In static conditions (FSS 0), Claudin-2 was detected in both plasma membrane and cytoplasm (as dot-like structures) while ZO-1 was only localized at the plasma membrane majorly as a continuous belt (Fig 1A). FSS caused the disappearance of Claudin-2 and changes the staining of ZO-1, which remained at the plasma membrane but becomes punctuated and discontinuous (Fig 1A). These modifications were concomitant with downregulation of protein expression, as revealed by Western blot analysis (S1 Fig). These results indicate that FSS induces disruption of tight junctions in tubular cells. The integrity of Par complex, that plays a critical role in tight junction formation and epithelial polarization [36, 37], was also investigated by measuring expression of Pard6 (Partitioning defective-6), Pard3 (Partitioning defective-3), aPKC (atypical protein kinase C) and Cdc42 (cell division cycle 42). When HK-2 cells were subjected to FSS, no change in mRNA levels of Pard3, aPKC and Cdc42 was detected (Fig 1B). However Pard6 mRNA expression was strongly reduced (Fig 1B), suggesting that FSS induces disruption of the Par complex thereby strengthening the demonstration that FSS leads to tight junction alteration.

**Fig 2:**
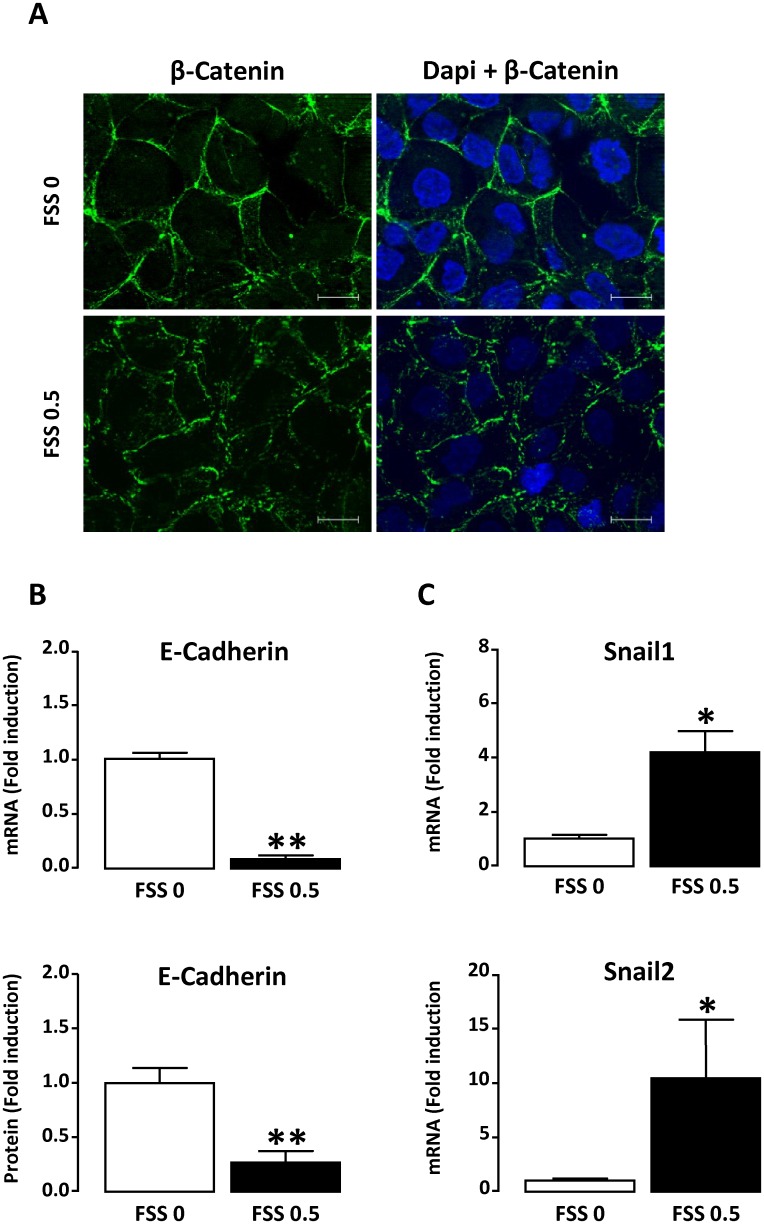
Alteration of adherens junctions in response to FSS. Confluent monolayers of HK-2 cells were submitted to FSS 0 (static) or FSS 0.5 Pa (FSS 0.5) for 48h. **A/** Immunofluorescence detection of β-Catenin. Cells were counterstained with DAPI. Pictures display representative areas of staining from three independent experiments. Green, β-Catenin; blue, DAPI-nuclei. Bar indicates 20 μm. **B/** Real-time PCR and Western blot analysis of E-Cadherin mRNA and protein, respectively. **C/** Real-time PCR was used for evaluation of mRNA levels encoding Snail1 or Snail2. In B and C, results are expressed as the fold induction compared to static condition and data represent mean ± SEM of 4–7 experiments. *p<0.05, **p<0.01 *versus* FSS 0.

The effect of FSS on adherens complexes was studied by analyzing by immunocytochemistry the localization of β-Catenin and by quantifying the expression of E-Cadherin as well as two of its repressor transcription factors, Snail1 and Snail2. In response to FSS, although staining is still seen predominantly in the periphery of the cell, β-catenin distribution was disrupted, with a more jagged and discontinuous pattern (Fig 2A). This effect was not accompanied with significant change in β-Catenin protein expression (S1 Fig). In parallel, mRNA and protein E-Cadherin expression was significantly reduced (Fig 2B) whereas the level of mRNA encoding Snail1 and Snail2 was significantly increased (Fig 2C). These results indicate that FSS disturbs adherens junctions of tubular cells.

To test the influence of FSS intensity, HK-2 cells were subjected to FSS 0 (static control), 0.01, 0.1 or 0.5 Pa for 48h and a number of epithelial cell specific markers were investigated as described previously (S2 Fig). In response to FSS 0.01 Pa, staining of Claudin-2, ZO-1 and β-Catenin was not modified. Claudin-2 expression strongly decreased with increasing FSS and staining of ZO-1 was found with a punctuated pattern in the plasma membrane under FSS 0.1 Pa and exacerbated at a FSS of 0.5 Pa. Finally, FSS 0.5 Pa induced disorganization of β-Catenin. The response of tubular cells to FSS appears thus to be dependent on the intensity of FSS applied.

### In Vitro Effect of FSS on Primary Cilia and Actin Cytoskeleton

**Fig 3:**
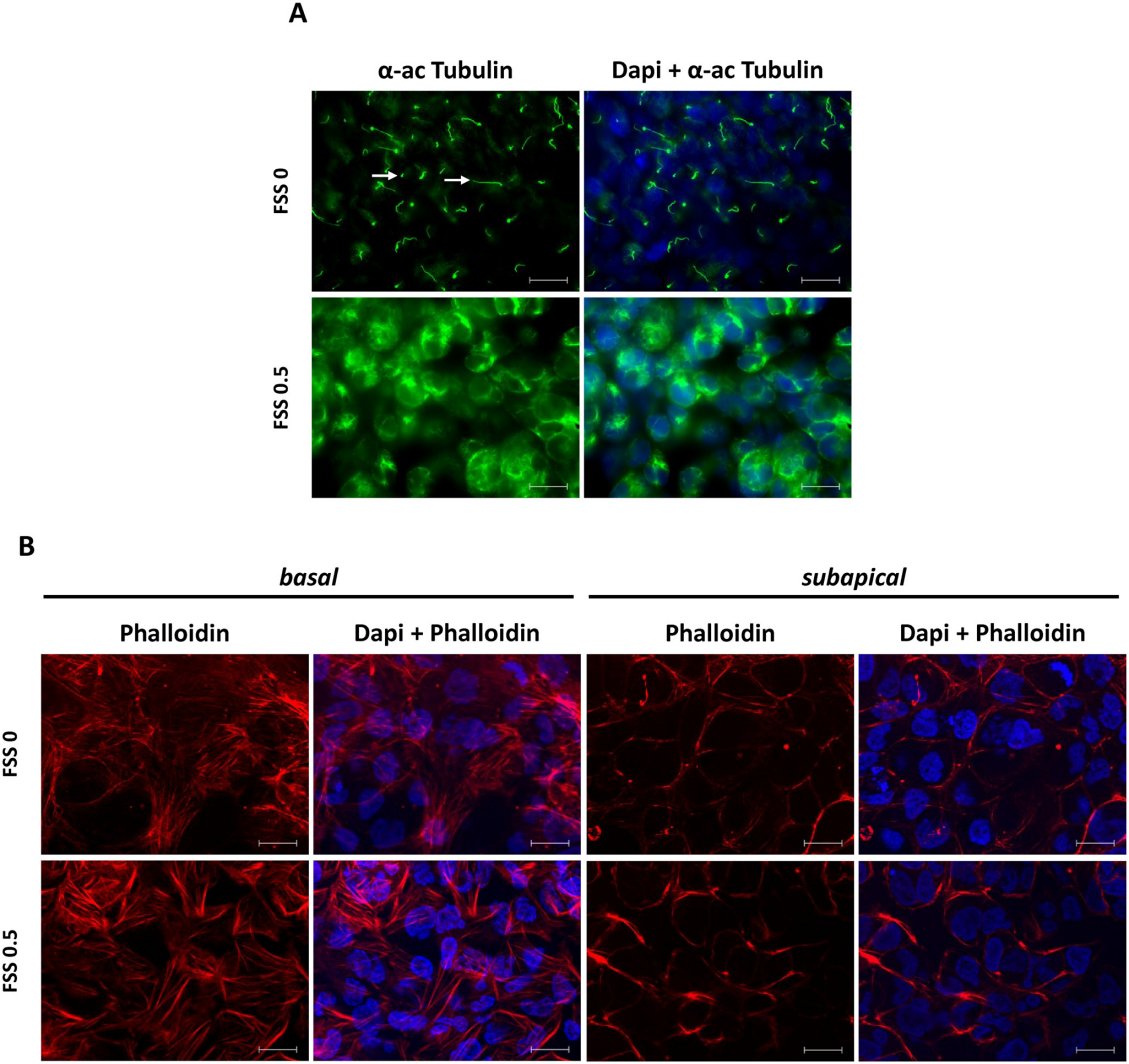
FSS-exposed cells exhibit loss of the primary cilium without marked change in actin cytoskeleton organization. Confluent monolayers of HK-2 cells were submitted to FSS 0 (static) or FSS 0.5 Pa (FSS 0.5) for 48h. **A/** α-acetylated Tubulin was analyzed by immunofluorescence to visualize the primary cilium. White arrows show primary cilia. **B/** Phalloidin was used to stain the actin cytoskeleton (basal [left] and subapical [right]). Cells were counterstained with DAPI. Pictures display representative areas of staining from 5 independent experiments. Green, α-acetylated Tubulin; red, Phalloidin; blue, DAPI-nuclei. Bars indicate 20 μm.

Next, we examined whether FSS (0.5 Pa, 48h) induces loss of the primary cilium in tubular cells. Cilia were visualized by immunostaining with anti α-acetylated Tubulin antibody. As shown in Fig 3A, cilia were observed in HK-2 cells in static conditions and FSS induced disappearance of cilia, accompanied by relocation of α-acetylated Tubulin into the cytoplasm. Since intercellular junctions and the cilium basis interact with actin cytoskeleton, we also studied the effect of FSS on the organization of the actin cytoskeleton in HK-2 cells. For this, we used Phalloidin staining, which detects filamentous (F) actin, and Apotome-imaging microscopy, to sequentially analyze basal and apical sides. In static conditions, actin was found as numerous long and thick cytosolic stress fibers spanning the entire cross sectional area of the cells at the basal side while actin microfilaments are organized as a thin circumferential network at cell-cell contacts at the subapical side (Fig 3B). Under FSS 0.5 Pa (48h), no marked change in actin microfilament organization was observed (Fig 3B), suggesting that FSS-treated cells did not display marked F-actin rearrangement. These results indicate that the perturbation of intercellular junctions and the deciliation of tubular cells generated by FSS are independent from F-actin rearrangement.

### In Vitro Effect of FSS on Cell Death and EMT

**Fig 4:**
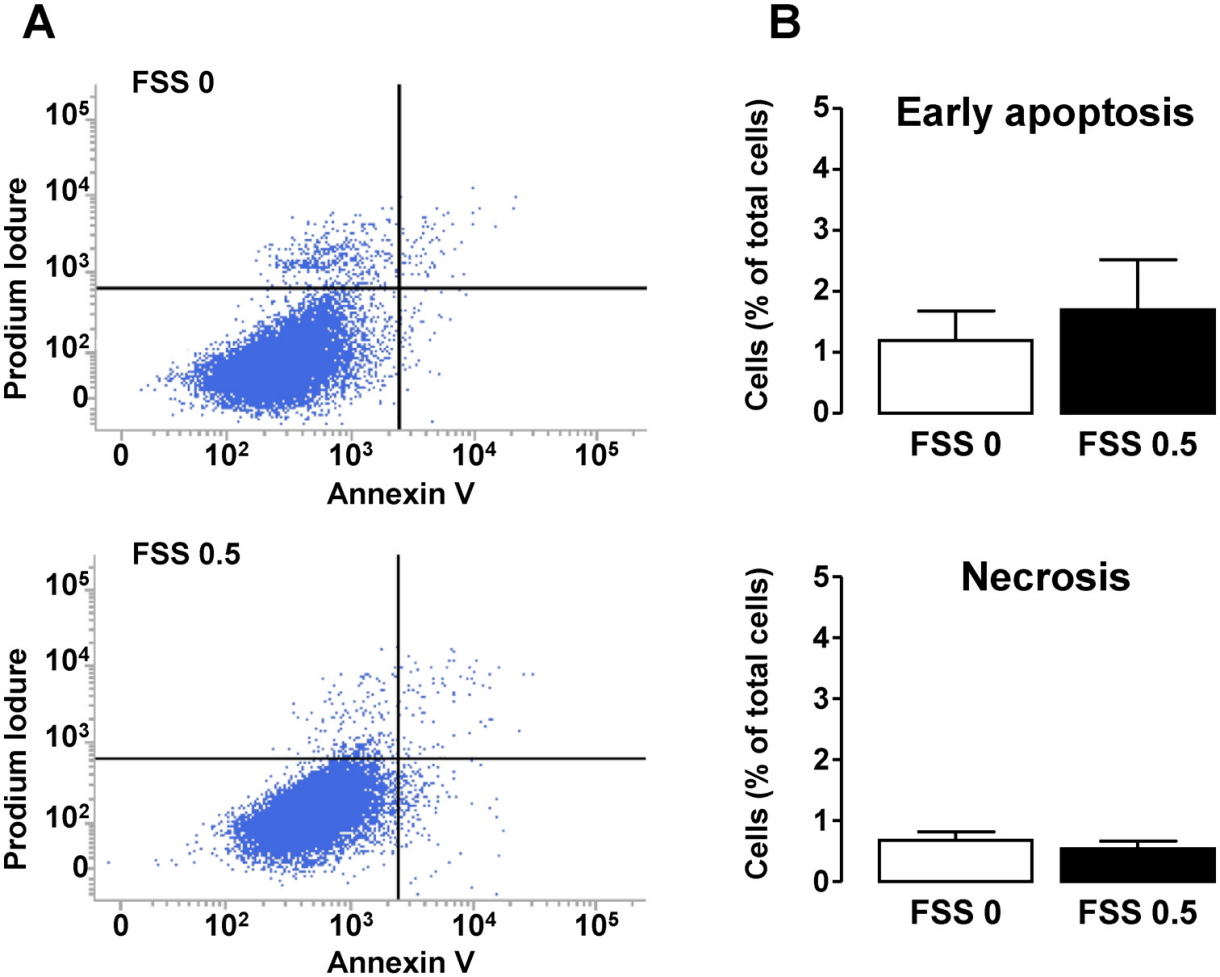
Effect of FSS on apoptosis and necrosis in tubular cells. Confluent monolayers of HK-2 cells were submitted to FSS 0 (static) or FSS 0.5 Pa (FSS 0.5) for 48h. **A/** Cells were stained with Annexin-V and then immediately subjected to analysis of phosphatidylserine externalization (Annexin-V fluorescence, X-axis) and Propidium Iodure (PI) uptake (PI fluorescence, Y-axis) using flow cytometry. Living, early apoptotic or necrotic (primary or secondary) cells were distinguished by the criteria of Annexin-V^−^/PI^−^(bottom left quadrant), Annexin-V^+^/PI^−^ (bottom right quadrant) and Annexin-V^+^/PI^+^ (upper right quadrant), respectively. **B**/ Proportions of early apoptosis and necrosis cells were quantified and results are expressed as a percentage of the total population of cells. Data represent mean ± SEM of 7 experiments.

Tubular apoptosis and necrosis are exacerbated in CKD, thereby contributing to tubular atrophy [38]. In addition, our laboratory has previously shown that exposure of HK-2 cells to FSS causes hyper-secretion of TNF-α [15], known to induce apoptosis in these cells [39]. To test whether FSS-induced dedifferentiation was associated to tubular cell death, HK-2 cells exposed or not to FSS 0.5 Pa for 48h were double-labeled with annexin V and propidium iodide. Analysis was performed by flow cytometry to separate live cells (non-labeled), cells in early phase of apoptosis (annexin V-positive, negative for propidium iodide) and necrotic cells (post-apoptotic or not, double positive). As shown in Fig 4, no change in the proportions of the different cell populations was observed between FSS 0 and FSS 0.5 Pa, thereby indicating that chronic FSS does not cause apoptosis or necrosis of the tubular cells.

**Fig 5:**
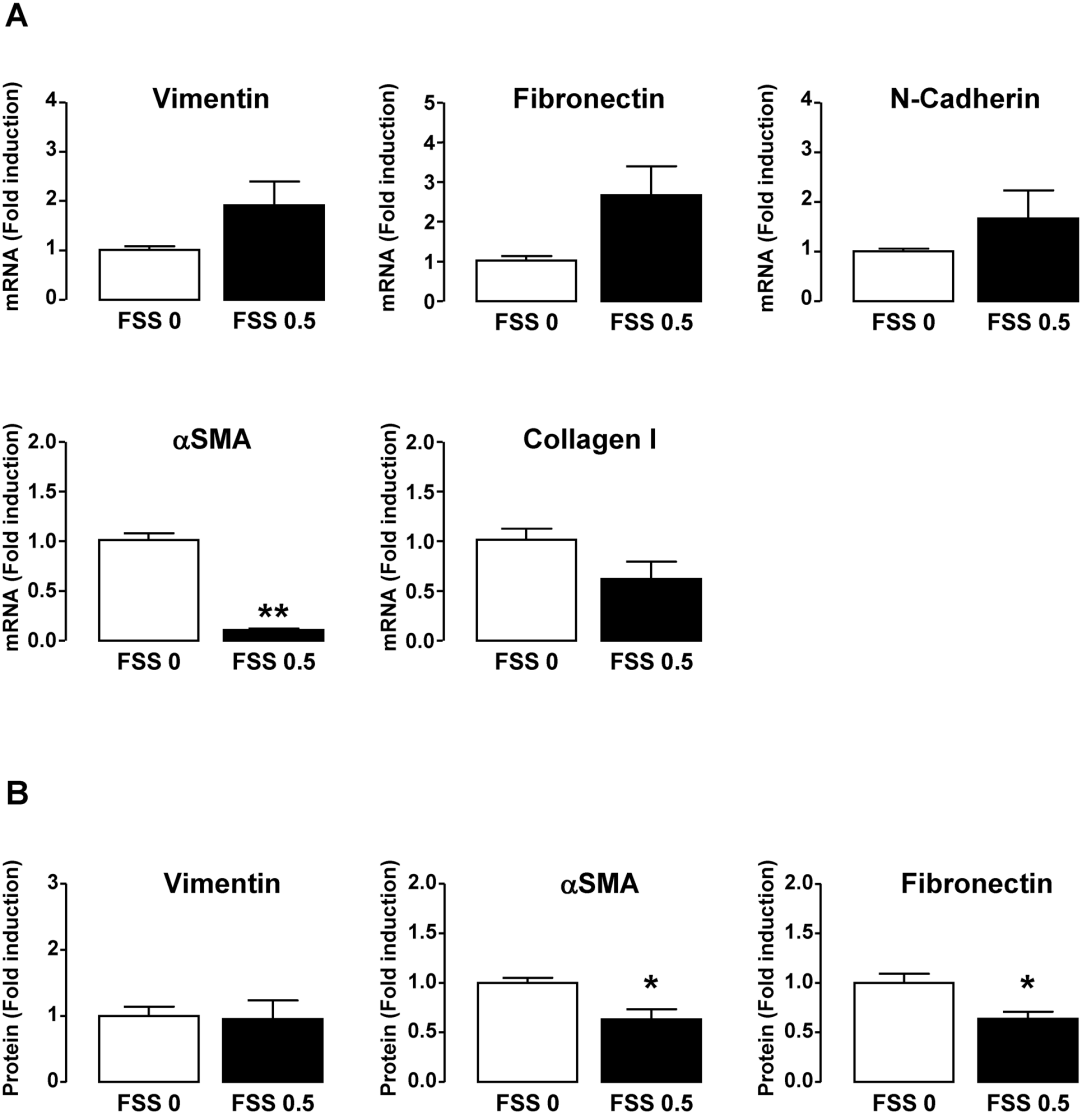
FSS does not induce a mesenchymal phenotype in tubular cells. Confluent monolayers of HK-2 cells were submitted to FSS 0 (static) or FSS 0.5 Pa (FSS 0.5) for 48h. **A**/ Transcript level of mesenchymal markers such as Vimentin and αSMA (cytoskeleton), Fibronectin and Collagen I (ECM), N-Cadherin (intercellular junction) was quantified by real-time PCR. Results are expressed as the fold induction compared to static condition. **B**/ Protein level of Vimentin, αSMA and Fibronectin was measured by Western blot. Results are expressed as the fold induction compared to static condition and data represent mean ± SEM of 4 experiments. *p<0.05, **p<0.01 *versus* FSS 0.

Even if the real contribution of EMT (epithelial mesenchymal transition) in renal fibrogenesis remains controversial, many studies showed that injured tubular cells lose epithelial features and acquire mesenchymal characteristics in CKD [38, 40]. To verify whether the above observed FSS-induced changes on epithelial cells were accompanied by an EMT, the expression of Vimentin, αSMA, Fibronectin, Collagen I and N-Cadherin as mesenchymal markers was measured in FSS subjected HK-2 cells. As shown in Fig 5A, FSS did not significantly change the level of mRNA encoding Vimentin, Fibronectin, Collagen I and N-Cadherin. Results were confirmed at protein level, as demonstrated for Vimentin and Fibronectin (Fig 5B). In addition, FSS induced a downregulation of αSMA mRNA (Fig 5A) but this was not confirmed at the protein level (Fig 5B). These results indicated that FSS does not lead to EMT.

### In Vivo Effect of Increased Urinary FSS

**Fig 6:**
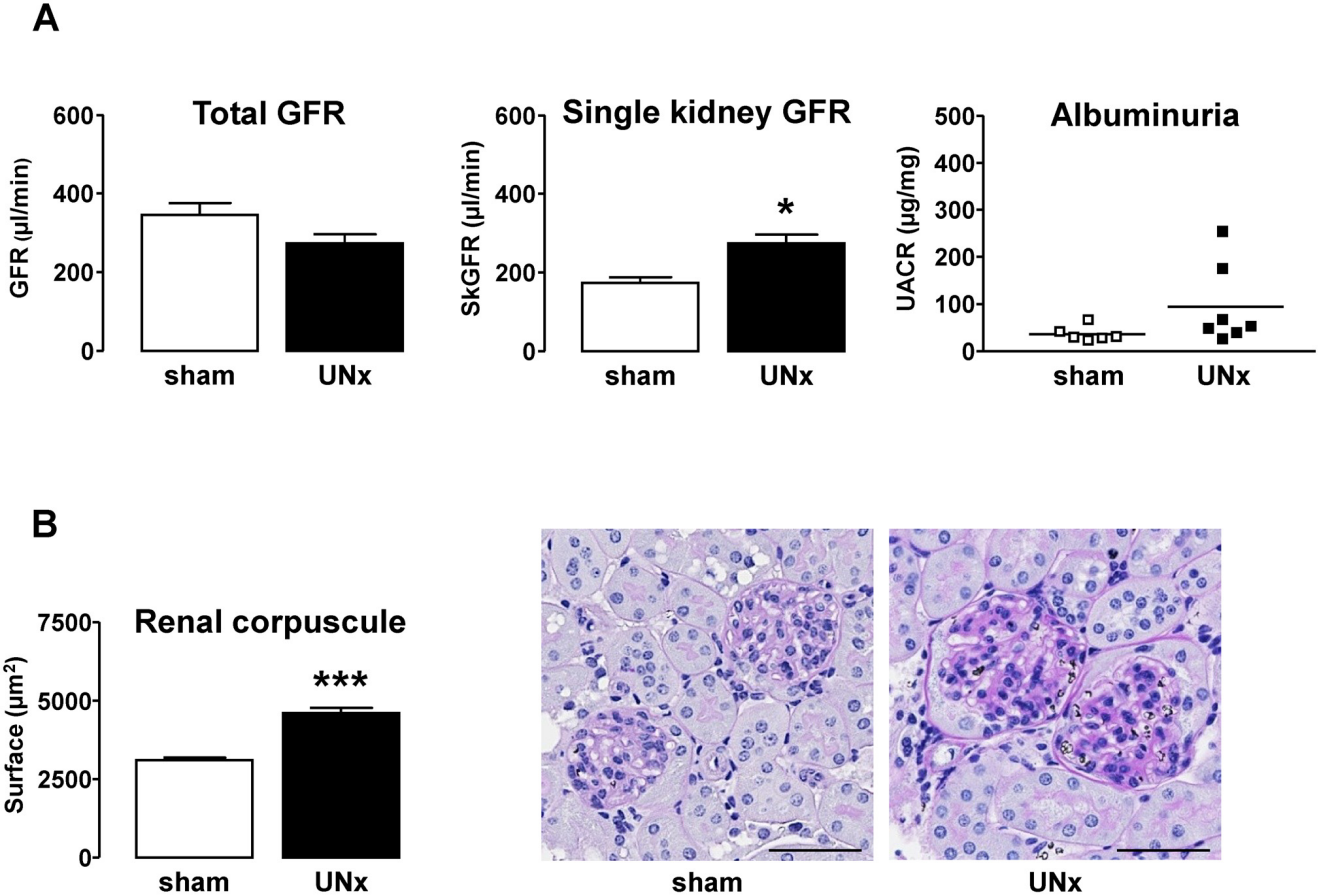
Uninephrectomy as an animal model of increased urinary FSS. Sham- and UNx-mice were analyzed 8 months after surgery. **A**/ Renal function was evaluated by measuring glomerular filtration rate (GFR), single kidney GFR (skGFR) and urinary albumin/creatinine ratio (UACR). **B**/ Renal corpuscule surface was measured on PAS-stained kidney slices. Pictures display representative areas of staining and bars indicate 200 μm. Data represent mean ± SEM from 6 animals per group. *p<0.05, ***p<0.01 *versus* sham.

**Fig 7:**
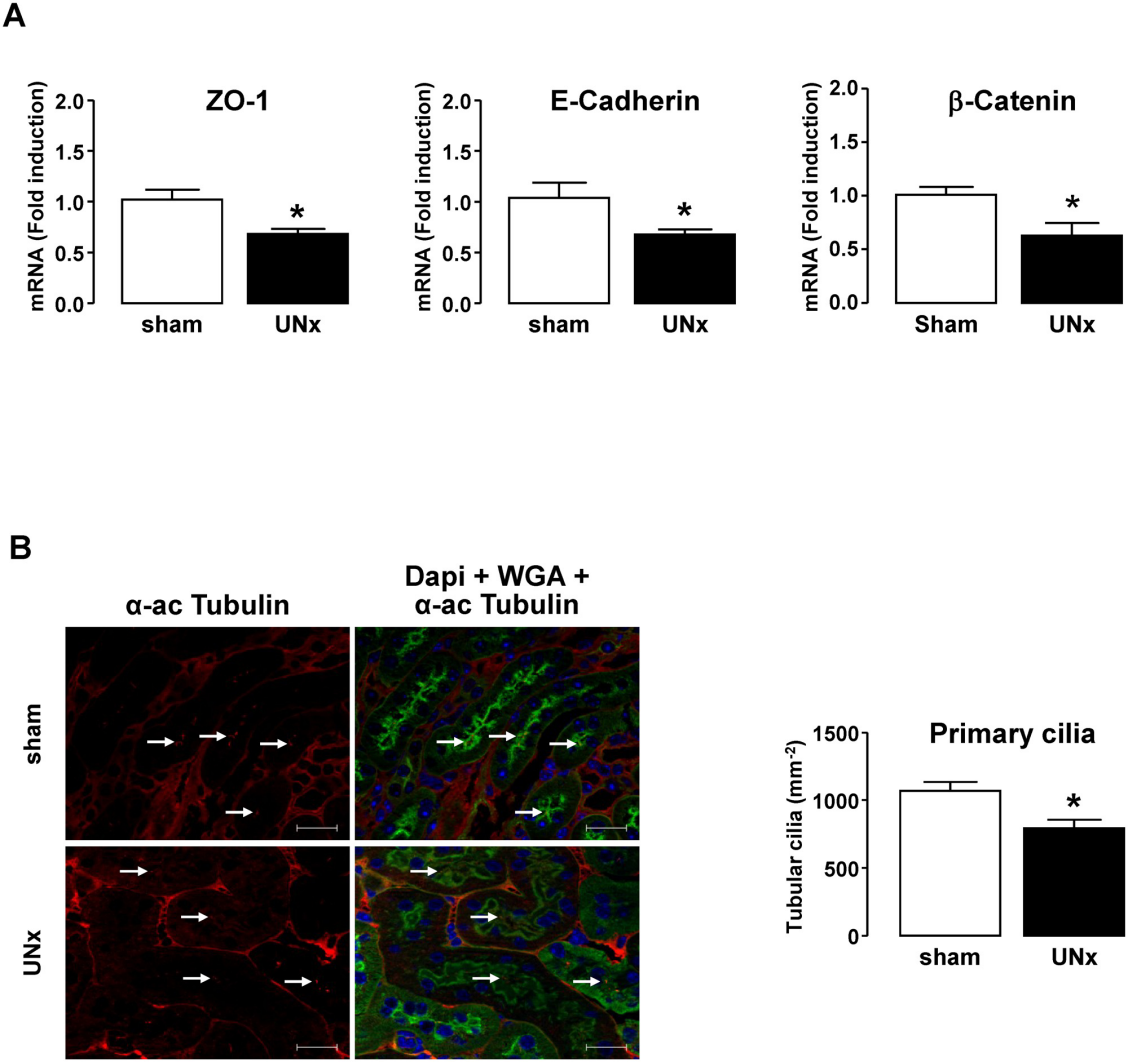
Effect of uninephrectomy-mediated FSS on epithelial gene expression and the density of primary cilia. Sham- and UNx-mice were analyzed 8 months after surgery. **A**/ The expression of ZO-1, E-cadherin and β-Catenin mRNA was quantified by real-time PCR from total RNA extracted from kidney cortex. Results are expressed as the fold induction compared to sham. **B**/ Immunofluorescence detection of α-acetylated Tubulin for quantification of the primary cilium. Kidney sections were counterstained with WGA and DAPI. Pictures in the left panel display representative areas of staining. Red, α-acetylated Tubulin; green, WGA-cell membranes; blue, DAPI-nuclei. Bar indicates 20 μm and white arrows show primary cilia. Graph in the right panel displays quantification of primary cilia by cortex tubular section. Data represent mean ± SEM from 6 animals per group. *p<0.05 *versus* sham.

Finally, we evaluated *in vivo* the effect of increased urinary FSS. We used an animal model where increased FSS was induced in proximal tubule by increased urinary flow following hyperfiltration. For this, C57BL/6 mice were uninephrectomized (UNx) by removing the right kidney. The left kidney was harvested 8 months later to analyse tubular epithelial markers. As expected [22–24], total GFR was maintained within the normal range in UNx subjected animals through adaptive increased single kidney (sk) GFR (Fig 6A), thereby leading to increased urinary FSS in remnant nephrons. In addition, elevated skGFR was accompanied by a significant glomerular hypertrophy, as indicated by increase of the renal corpuscule area (Fig 6B), thereby confirming the compensatory hyperfiltration. Urine albumin excretion was not significantly modified (Fig 6A) and tubular dilatation was not detected (Fig 6B). However the mRNA level of epithelial makers ZO-1, E-Cadherin and β-Catenin was significantly decreased in UNx animals compared to sham (Fig 7A). In addition, a decreased number of primary cilia in tubular cells was detected (Fig 7B). Taking into account the observations *in vitro*, these data suggest that increased FSS *in vivo* is associated, as well, with a reduction of expression of epithelial markers.

## Discussion

### Pathologic FSS Leads to Structural Changes in Renal Tubular Cells

The present study demonstrates that *in vitro* exposure of proximal tubular cells to high and chronic FSS (0.5 Pa, 48h) leads to significant structural cell changes. Firstly, Claudin-2, ZO-1 and Pard6, which account for tight junction organization, were lost. Secondly, β-Catenin localisation and E-Cadherin abundance, as markers of adherens junctions, were disrupted and decreased, respectively. The latter was due to a strong FSS induced-stimulation of Snail1 and Snail2, two transcription factors well known to repress E-Cadherin expression. Thirdly, the primary cilium disappeared under FSS. Since tight junctions, adherens junctions and cilium are hallmarks of epithelial cells, our data indicate that tubular cells lose characteristics of differentiated epithelial cells in response to pathologic FSS. The deciliation is likely the consequence rather than the cause of tubular dedifferentiation, since removal of primary cilium by treatment with hydrate chloral failed to mimic the FSS effects in static-renal cells (data not shown).

Although FSS is known to influence renal function [1–5], its precise impact on epithelial organization of renal cells remains poorly understood. Some reports propose that FSS favors the differentiation of tubular cells by promoting actin cytoskeleton rearrangement and formation of intercellular junctions [2, 7, 33, 41–45]. Another study suggests that FSS causes disruption of tight junctions in tubular cells [46]. Our results are in agreement with this last study. Several reasons might account for the discrepancies between reports. i) The impact of FSS on tubular cells might be different according to its intensity, as demonstrated in S1 Fig In our study, the intensity of FSS was higher (0.5 Pa) compared to those applied in the other reports (0.002–0.1 Pa) [2, 7, 33, 41, 43–45], all lower or equal to physiological FSS (Table 1). Although under the physiological cutoff of FSS, Friedrich et al study [46] have shown that FSS-induced loss of epithelial phenotype is dependent on FSS level. In addition, other effects have been reported to be dependent on the intensity of FSS, such as e.g. the inhibition of the fibrinolytic system in LLC-PK1 cells [7] or the reduction in motility of HK-2 cells [47]. ii) The effect of FSS might also depend on its duration. We used chronic FSS exposure (48h) whereas shorter exposure were used in the other reports (2h–24h) [2, 7, 33, 41, 43–45] (Table 1). Moreover, it has been already demonstrated that tubular cells can respond to a same stimulus in a biphasic manner, with a long term effect which is the opposite of that in short term [48, 49]. Interestingly a strong temporal and oscillatory regulation of key Erk pathway molecules, Snail1 and Ocludin (a marker of tight junctions) has been identified in HK-2 cell exposed to FSS [47]. iii) The impact of FSS on tubular cells might also be different according to the species (human in our study *vs* mouse [7, 33, 43], rat [2, 44], dog [41] or pig [7]) and/or the type of renal cells (proximal tubule in our study *vs* collecting duct [2, 41, 44] or heterogeneous tubular cells [50]) (Table 1) since it is known that cells respond to FSS in a cell type-specific fashion [7, 33, 51]. iv) The type of culture cell substrate (material and ECM coating) (Table 1) appeared to be critical for the response to FSS [42, 45]. v) Finally, the probably most important difference between reports concerned the state of differentiation of renal cells before exposure to FSS. Cells that sense and respond to extracellular fluid flow have special sensory organelles such as primary cilium, glycocalyx layer, brush border microvilli [51] which are uniquely present in differentiated cells. Studies in [33, 41, 43–45, 50] were conducted on cells exhibiting no tight or adherens junctions nor primary cilium under static conditions, showing their low level of differentiation. In contrast, we and works in [46, 52] used more differentiated renal cells with already formed intercellular junctions in static conditions and it is known that the sensitivity of many cells to insults strongly depends on their differentiation state [53–55].

### Pathologic FSS Does Not Lead to Tubular Cell Death or EMT

Our data show that although FSS leads to structural changes of tubular cells, it is neither associated to cell death nor to EMT. Another mechanism that might lead to loss of functional epithelial cells is cell senescence. Cell senescence is a general stress-response program, characterized by an irreversible growth arrest and set of functional and morphological changes, especially including disrupted cell-cell junctions [56, 57]. Acceleration of senescence represents a relevant mechanism by which tubular cells are injured in diabetic nephropathy [58, 59] and FSS was proposed to contribute to senescence of chondrocytes and endothelial cells [60, 61]. Further investigations could therefore be conducted to test whether renal tubular cells adopt a senescence-like phenotype in response to FSS.

### Downstream Signals of FSS in Tubular Cells

We have previously observed that FSS exposure alters the secretion of a number of chemokines/cytokines in proximal tubular cells [14] that could account for the observed tubular changes. For example, FSS stimulated HK-2 cells release epidermal growth factor, a molecule that is known to induce disruption of tight and adherens junctions in renal tubular cells [62–64]. Nevertheless, several other scenarios downstream of FSS can be proposed. Wnt signaling, strongly associated with cell dedifferentiation, is activated by FSS in osteoblasts [65]. After stimulation, this pathway leads to nuclear translocation of β-Catenin, which in turn acts as a transcription factor. Our result show that FSS led to disorganization of β-Catenin staining at the periphery of the cells. However, there is no evidence to support migration of this protein into the nucleus, indicating that the Wnt pathway is probably not activated by FSS in renal tubular cells. Further experiments should be performed to definitively exclude a role of this signaling pathway. The hedgehog pathway is also associated with cell dedifferentiation because its inhibition in stomach epithelial cells causes disruption of intercellular junctions [66]. However, we found that blocking the hedgehog pathway in static HK-2 cells with cyclopamine did not mimic the FSS effects (data not shown), suggesting that hedgehog pathway is not responsible for the dedifferentiation under FSS.

### In Vivo Relevance of FSS Effects

Many pathological states directly affect intercellular junctional complexes [18, 19, 67–69]. In addition, significant shortening of cilia on proximal tubule cells, associated with cell dedifferentiation, was also observed in renal injury induced by ischemia-reperfusion [70] and in proximal tubular cells treated *in vitro* with transforming growth factor-beta (a key player in tubular aggression during CKD) [71]. Thus our *in vitro* results suggest that chronic non physiological FSS can be considered as an insult for proximal tubular cells, leading to significant structural changes of these cells. We obtained evidence for the role of FSS in the structural epithelial changes *in vivo*. The loss of nephron mass with resultant increased single nephron GFR has been recognized as a principal mediator that contributes to CKD progression [23, 24]. In our study, an UNx for 8 months induced hyperfiltration in the remnant kidney, thereby leading to increased rate of urine flow in the lumen of proximal tubule. FSS depends on the fluid flow rate and viscosity as well as on the diameter of the tube. Since there was no notable change in tubular diameter, the increased skGFR most likely induced increased urinary FSS in the proximal tubule in this model. A decrease in epithelial gene expression such as ZO-1 and E-cadherin was observed concomitantly with elevated skGFR. As this was observed in absence of other mediators of tubular aggression (such as albuminuria, dilatation-induced stretch or hyperglycemia (data not shown)), our result strongly supports an *in vivo* role of pathologic FSS in tubular lesions after reduction in nephron number. Glomerular hyperfiltration has been incriminated as a common pathogenic mechanism leading to CKD progression in other conditions including diabetes mellitus, hypertension, obesity, polycystic kidney disease, sickle cell anemia, or the nephrotic syndrome [72, 73]. It is therefore tempting to propose that tubular lesions induced by hyperfiltration in these situations may be caused, at least in part, by increased urinary FSS and its ability to trigger structural changes of renal tubular cells.

In conclusion, the present study demonstrates that proximal tubular cells lose an important number of their epithelial characteristics, such as tight junctions, adherens junctions and primary cilium, after long term exposure to FSS both *in vitro* and *in vivo*. Thus, the changes in FSS induced by variations of urinary fluid flow and urine composition should be considered as potential insults for tubular cells leading to disorganization of the tubular epithelium. Since modified urinary FSS occurs in early phases of most nephropathies, increased FSS can thus contribute to the (primary) induction of the tubular lesions. Further studies on the mechanisms associated to FSS-induced alterations would be of great interest to propose new targets to slow-down progression of CKD.

## Supporting Information

S1 Fig Effect of FSS on epithelial junction proteins. Confluent monolayers of HK-2 cells were submitted to FSS 0 (static) or FSS 0.5 Pa (FSS 0.5) for 48h. The expression of Claudin-2, ZO-1 and β-Catenin protein was quantified by Western blot. Results are expressed as the fold induction compared to static condition and data represent mean ± SEM of 3–5 experiments. *p<0.05, **p<0.01 *versus* FSS 0.
(TIF)

S2 Fig Effect of FSS intensity. Confluent monolayers of HK-2 cells were maintained to FSS 0 (static) or subjected to FSS 0.01 Pa (FSS 0.01), 0.1 Pa (FSS 0.1) or 0.5 Pa (FSS 0.5) for 48h. The localization of Claudin-2, ZO-1 or β-Catenin was analyzed by immunofluorescence. Pictures display representative areas of staining from three independent experiments. Bars indicate 20 μm.
(TIF)
