# Supplementary figures and images for: Shear Stress-Induced Alteration of Epithelial Organization in Human Renal Tubular Cells
Source: PLoS One. 2015 Jul 6;10(7):e0131416. doi: 10.1371/journal.pone.0131416 (PMC4493045; doi:10.1371/journal.pone.0131416)

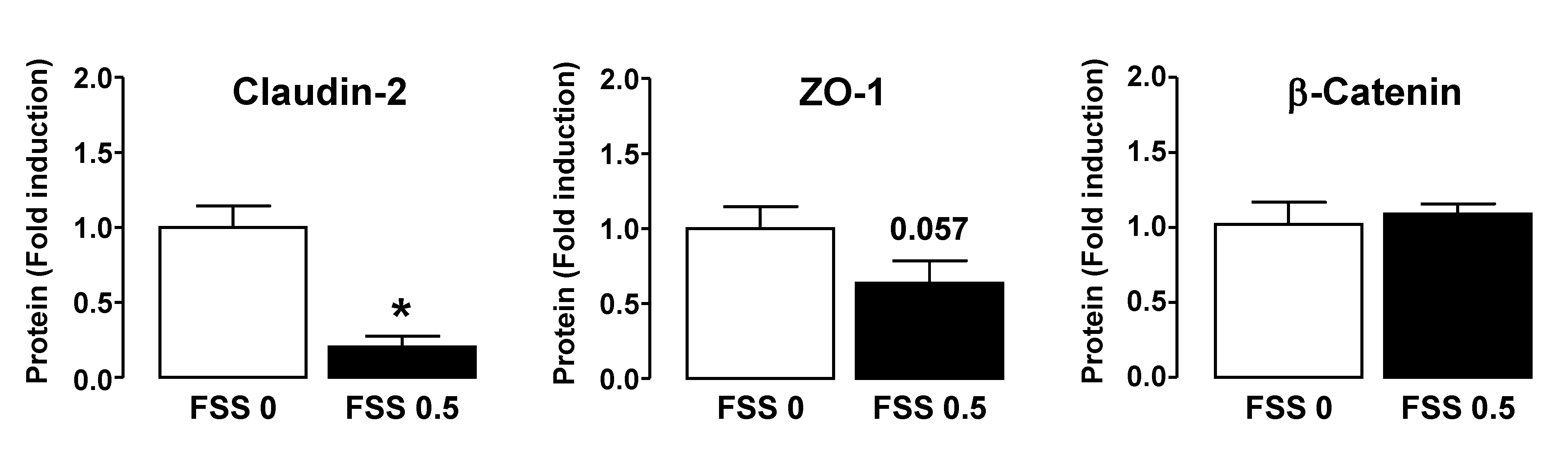

Supplement: S1 Fig — Confluent monolayers of HK-2 cells were submitted to FSS 0 (static) or FSS 0.5 Pa (FSS 0.5) for 48h. The expression of Claudin-2, ZO-1 and β-Catenin protein was quantified by Western blot. Results are expressed as the fold induction compared to static condition and data represent mean ± SEM of 3–5 experiments. *p<0.05, **p<0.01 versus FSS 0. (TIF) [file pone.0131416.s001.tif]

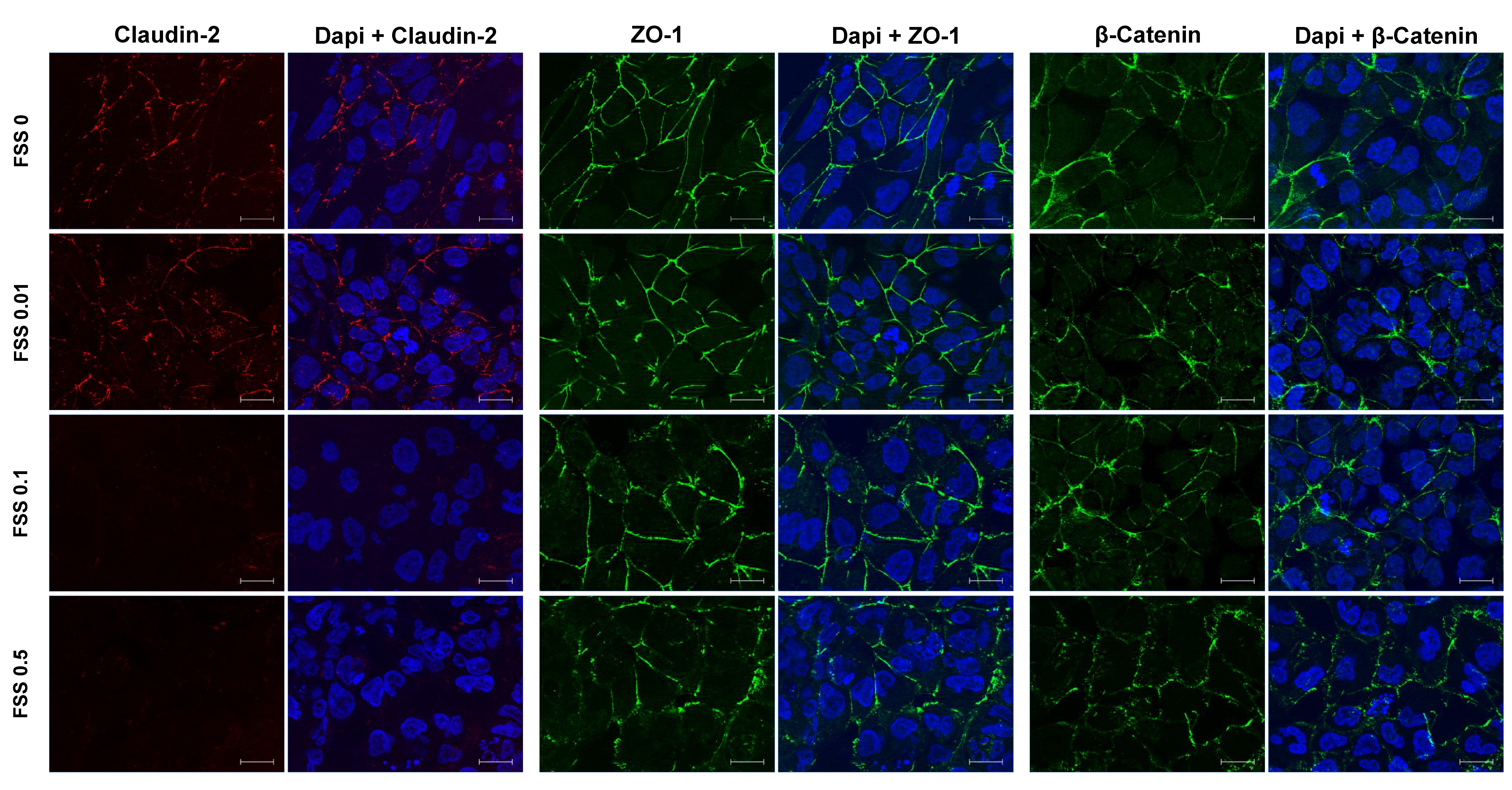

Supplement: S2 Fig — Confluent monolayers of HK-2 cells were maintained to FSS 0 (static) or subjected to FSS 0.01 Pa (FSS 0.01), 0.1 Pa (FSS 0.1) or 0.5 Pa (FSS 0.5) for 48h. The localization of Claudin-2, ZO-1 or β-Catenin was analyzed by immunofluorescence. Pictures display representative areas of staining from three independent experiments. Bars indicate 20 μm. (TIF) [file pone.0131416.s002.tif]
